# IDH1^R132H^ in Neural Stem Cells: Differentiation Impaired by Increased Apoptosis

**DOI:** 10.1371/journal.pone.0154726

**Published:** 2016-05-04

**Authors:** Kamila Rosiak, Maciej Smolarz, Wojciech J. Stec, Joanna Peciak, Dawid Grzela, Marta Winiecka-Klimek, Ewelina Stoczynska-Fidelus, Barbara Krynska, Sylwester Piaskowski, Piotr Rieske

**Affiliations:** 1 Department of Tumor Biology, Medical University of Lodz, Zeligowskiego 7/9, 90–752, Lodz, Poland; 2 Department of Research and Development, Celther Polska, Milionowa 23, 93–193, Lodz, Poland; 3 Shriners Hospitals Pediatric Research Center, Center for Neural Repair and Rehabilitation, Temple University School of Medicine, 3500 N. Broad Street, Philadelphia, PA, 19140, United States of America; University of Colorado, Boulder, UNITED STATES

## Abstract

**Background:**

The high frequency of mutations in the isocitrate dehydrogenase 1 (IDH1) gene in diffuse gliomas indicates its importance in the process of gliomagenesis. These mutations result in loss of the normal function and acquisition of the neomorphic activity converting α-ketoglutarate to 2-hydroxyglutarate. This potential oncometabolite may induce the epigenetic changes, resulting in the deregulated expression of numerous genes, including those related to the differentiation process or cell survivability.

**Methods:**

Neural stem cells were derived from human induced pluripotent stem cells following embryoid body formation. Neural stem cells transduced with mutant IDH1^R132H^, empty vector, non-transduced and overexpressing IDH1^WT^ controls were differentiated into astrocytes and neurons in culture. The neuronal and astrocytic differentiation was determined by morphology and expression of lineage specific markers (MAP2, Synapsin I and GFAP) as determined by real-time PCR and immunocytochemical staining. Apoptosis was evaluated by real-time observation of Caspase-3 activation and measurement of PARP cleavage by Western Blot.

**Results:**

Compared with control groups, cells expressing IDH1^R132H^ retained an undifferentiated state and lacked morphological changes following stimulated differentiation. The significant inhibitory effect of IDH1^R132H^ on neuronal and astrocytic differentiation was confirmed by immunocytochemical staining for markers of neural stem cells. Additionally, real-time PCR indicated suppressed expression of lineage markers. High percentage of apoptotic cells was detected within IDH1^R132H^-positive neural stem cells population and their derivatives, if compared to normal neural stem cells and their derivatives. The analysis of PARP and Caspase-3 activity confirmed apoptosis sensitivity in mutant protein-expressing neural cells.

**Conclusions:**

Our study demonstrates that expression of IDH1^R132H^ increases apoptosis susceptibility of neural stem cells and their derivatives. Robust apoptosis causes differentiation deficiency of IDH1^R132H^-expressing cells.

## Introduction

Diffusely infiltrating gliomas are the most common tumours of the central nervous system [1]. Despite the multimodal treatment strategies comprising neurosurgical resection, radiotherapy and chemotherapy, these neoplasms have an inherent tendency towards recurrence and progression [2,3]. Gliomas comprise a heterogeneous group of neoplasms with unknown causes and not fully elucidated mechanisms of development. The recent high-throughput analyses by Eckel-Passow *et al*. and TCGA Research Network thoroughly described the specific patterns of molecular alterations in gliomas, which correlated with the histopathological subtypes and clinical presentation [4,5]. The most common mutations affect the genes coding isocitrate dehydrogenases: IDH1 (cytosolic) and IDH2 (mitochondrial) and are detected in about 70–80% of grade II/III gliomas (and secondary glioblastomas, grade IV) [6]. Therefore, these mutations appear as an essential and initial step in gliomagenesis [7]. Over 90% of *IDH1* mutations involve substitution of arginine by histidine in the enzyme’s active site at codon 132 (R132H) [8]. Physiological function of IDH1 in all cells is to catalyse oxidative decarboxylation of isocitrate (with the formation of alpha-ketoglutarate, α-KG), which is one of the most important sources of NADPH. Thus, it is vital for the maintenance of the proper oxidation-reduction potential and the antioxidative protection of cells [9,10]. In addition to the disruption of the enzyme function, this mutation also results in the acquisition of a neomorphic activity, transforming α-KG to 2-hydoxyglutarate (2-HG), which is considered an oncometabolite [11]. Both the decrease in α-KG and the increase in 2HG cellular concentrations affect the activity of numerous dioxygenases, including prolyl hydroxylases as well as chromatin modifying enzymes (*e*.*g*. histone demethylases and TET family proteins) [12], which regulate the expression of various genes and may be involved in the process of differentiation, one of the major parameters of malignant cell transformation.

Although the origins of glioma are not well understood, gliomas are generally thought to arise from the neural stem cells or the populations of progenitor cells [13]. The primary goal of the present project was to assess the differentiation potential of the neural stem cells derived from the human induced pluripotent stem cells, that stably express the mutant IDH1 gene harboring a single point R132H mutation. At the same time the effect of IDH1^R132H^ on the apoptosis sustainability in the studied cells was scrutinized. In this study we propose a new model system of human neural stem cells to investigate influence of IDH1^R132H^ mutation on neural stem cells and their derivatives.

## Materials and Methods

### Cell culture

The ebiNSc, a human induced neural stem cell line obtained from induced pluripotent stem cells following embryoid bodies generation and induction of neural differentiation, and ebiNSc^IDH1R132H^, a human cell line with an induced expression of mutated IDH1^R132H^ gene, were gifts received from Celther Polska Ltd. The ebiNSc were obtained as described previously [14]. The ebiNSc^IDH1WT^ and ebiNSc^empty^ were generated, analogously to ebiNSc^IDH1R132H^, *via* the transduction with the respective vector (as described below).

In order to ensure the reliability of the results, we employed four independently generated populations of ebiNSc. All ebiNSc cell lines were propagated as an adherent culture on Geltrex (Life Technologies, US) coated dishes in neural stem cell maintenance medium (self-renewal conditions; ReNcell medium, Merck Millipore, Germany, supplemented with 20 ng/mL bFGF and 20 ng/mL EGF, both Sigma, US). Cells were cultured at 37°C in 5% CO_2_, 95% humidity, and without O_2_ control.

### Construction of a lentiviral vector expressing IDH1^WT^

The IDH1 gene was amplified with primers containing specific Gateway® att cloning sites: 5’- ggggacaagtttgtacaaaaaagcagcgtatgtccaaaaaaatcagtggcg -3’ (forward) and 5’- ggggaccactttgtacaagaaagctgggttaaagtttggcctgagctagt -3’ (reverse). PCR products were cloned into pENTR^TM^/Zeo vector and subsequently transferred to pLEX_307 plasmid (Addgene, US) using Gateway® Cloning Technology (Life Technologies) according to the manufacturer's protocol. Following successful construction, confirmed by direct sequencing, lentiviral vector carrying cDNA of IDH1^WT^ was prepared using LENTI-Smart™ (InvivoGen, US) following the manufacturer's recommendations. Briefly, 24h before transfection, 5x10^6^ HEK293T cells were seeded in the 10 cm dish and cultured in DMEM High Glucose (Biowest, France) supplemented with 10% FBS (Biowest). On the following day, the transfection complex was added. After 24 hours, the cell culture medium was changed. After the next two days the medium was collected and subsequently filtered through a 0.45 μm filter (Merck Millipore) and stored at -80˚C. Empty lentiviral vector was obtained analogously, without inserted sequence.

### Lentiviral transduction of Neural Stem Cells

For the generation of ebiNSc cell line with stable expression of empty vector or wild type *IDH1*, the cells were seeded at 5x10^5^ per well and transduced with the proper lentiviral vector. After 48 hours, the medium was changed and the cells were incubated in neural stem cell maintenance medium with the addition of puromycin (2.5 μg/mL; InvivoGen) for 7 days. Pooled populations of puromycin resistant cells were obtained and cultured continuously with puromycin (1 μg/mL).

### Induction of neuronal and astrocytic differentiation

All ebiNSc cell lines were seeded into Geltrex coated 4-well plates at 2.5×10^4^ cells/well, and grown in medium for neural stem cell maintenance. Two days after seeding, the medium was changed into neural differentiation medium (Neurobasal Medium 1X, B-27 Serum Free Supplement 2%, GlutaMAX-I Supplement 2 mM; all from Life Technologies). Half of the medium was changed every 2–3 days.

The described method is intended primarily for the preparation of neuronal cells. Nevertheless, it is also possible to obtain a population of astrocytes in these conditions [15].

### Immunocytochemical analysis

Immunocytochemical stainings were performed after 0, 7 and 14 days of ebiNSc differentiation, as previously described [16]. Briefly, cells were fixed in 4% paraformaldehyde and permeabilized with 0.1% Triton X-100. Non-specific binding sites were blocked with 2% donkey serum (Sigma). The fixed cells were subsequently incubated overnight with appropriate primary antibodies (Table 1). After washing, cells were incubated with secondary antibodies for 1 hour at room temperature. The slides were mounted with ProLong^®^ Gold Antifade Reagent with DAPI (Molecular Probes), coverslipped, and imaged on fluorescent microscope (MN-800 FL, OPTA-TECH, Poland).

**Table 1:** Antibodies used for immunocytochemical staining.

Antibody	Host	Manufacturer	Dilution
anti-IDH1 R132H	mouse	Dianova, DIA-H09	1: 50
anti-IDH1 (D2H1)	rabbit	Cell Signaling Technology, Inc., 8137	1: 400
anti-SOX2	rabbit	Abcam, ab97959	1:500
anti-MAP2	rabbit	Abcam, ab32454	1: 500
anti-GFAP (GA5)	mouse	Merck Millipore, MAB360	1: 800
anti-Synapsin I	rabbit	Merck Millipore, AB1543	1: 500
anti-Nestin	mouse	Santa Cruz Biotechnology, sc-71665	1:500
anti-mouse Alexa Fluor^®^594	donkey	Molecular Probes, Invitrogen	1: 500
anti-rabbit Alexa Fluor^®^488	donkey	Molecular Probes, Invitrogen	1: 500

### Western Blot

Cells were lysed in RIPA buffer (Sigma) supplemented with Protease Inhibitor Cocktail (Sigma). Equal amounts of total proteins (20ug) were separated on 8% SDS–polyacrylamide gel and transferred onto PVDF membranes (Immobilon—P, Merck Millipore). Subsequently, membranes were blocked with 5% skim milk (Sigma) and incubated overnight at 4°C with primary antibodies (Table 2). Bands were visualized with enhanced chemiluminescence (Amersham ECL Prime Western Blotting Detection Reagent, GE Healthcare).

**Table 2:** Antibodies used in Western Blot.

Antibody	Host	Manufacturer	Dilution
anti-IDH1 R132H	mouse	Dianova, DIA-H09	1: 500
anti-IDH1 (D2H1)	rabbit	Cell Signaling Technology, Inc., 8137	1: 1000
anti-PARP	rabbit	Cell Signaling, 9542	1: 1000
anti-Actin, clone C4	mouse	Merck Millipore, MAB1501	1: 4000
anti-rabbit IgG-HRP	goat	Santa Cruz Biotechnology, Inc., sc-2004	1: 4000
anti-mouse IgG-HRP	goat	Santa Cruz Biotechnology, Inc., sc-2005	1: 4000

### Light microscopy and real-time cell observations

Light microscopy observations of cells differentiation were performed using inverted microscope (MW100, OPTA-TECH) with pictures taken every 24 hours for 14 days (at magnification 40x).

Real-time cell monitoring of apoptosis was performed with the use of CellEvent™ Caspase-3/7 Green Detection Reagent (Molecular Probes, Invitrogen) and integrated cell culture observation device Biostation CT (Nikon Corporation, Tokyo, Japan) with images taken every 2 hours for 2 days (at magnification 100x).

### RNA isolation and Quantitative Real time PCR

Total RNA was extracted using AllPrep DNA/RNA Mini Kit (Qiagen). 200 ng of RNA was reverse transcribed to cDNA using QuantiTect Reverse Transcription Kit (Qiagen). Both procedures were performed according to the manufacturer’s protocols.

Quantitative RT-PCR was performed using StepOnePlus Real-Time PCR System (Applied Biosystems, US). Each sample was amplified in triplicate in total reaction volume of 12 μL containing SYBR^®^ Select Master Mix (2X) (Life Technologies), 200 nM of both forward and reverse primers and 200 ng of cDNA. The *HPRT1* gene was used as the reference gene to normalise the expression levels of the target gene. Specific primers were used for amplification of the tested genes (Table 3). The cycling conditions were as follows: 2 min at 50°C (UDG activation), 10 min at 95°C (polymerase activation) followed by 40 cycles of: 15 s at 95°C (denaturation), 30 s at 60°C (annealing), and 30 s at 72°C (extension).

**Table 3:** Primers sequences.

Gene	Sense primer	Antisense primer
*IDH1*	5'- ACATGGTGGCCCAAGCTA—3'	5'- AGCAATGGGATTGGTGGA -3'
*GFAP*	5'- GAGATCCGCACGCAGTATGA -3'	5'- CTTCAGGTCTGGCAGTGGTT -3'
*MAP2*	5'- CTATCCCAGGACCCCTCACA -3'	5'- CTTCAGGTCTGGCAGTGGTT -3'
*SYN1*	5’-GAAACCCAGCCAGGACGTG-3’	5’- GCTCTGGAAGGTTGAAGGCA -3’
*HPRT1*	5’- GACCAGTCAACAGGGGACAT -3’	5’- AACACTTCGTGGGGTCCTTTTC -3’

To confirm the specificity of the amplification signal, the gene dissociation curve was verified in each case. The normalised relative expression level of the analysed genes was calculated with the method described by Pfaffl *et al*. [17], based on sample’s average Ct value and gene’s average PCR efficiency (calculated with LinReg software). No Template Control (NTC) reaction was used to exclude PCR contamination. A diluted mixture of cDNA from all tested samples was used for the normalisation.

### Statistical analysis

Each experiment was performed three times. All statistical analyses were performed using GraphPad Prism 5 (Graphpad Software). The tests performed for each experiment are named in the figure legends for each experiment individually.

For the quantitative assessment of differentiation markers, cells were counted at 200x magnification in ten random fields per experiment (three experiments for each analysis).

Due to the morphology of differentiating cells (formation of clusters and networks) and the sequence of marker emergence, the counting was performed at day 7 for GFAP and MAP2 and at day 14 for Synapsin I. The proportion of GFAP^+^, MAP2^+^ and Synapsin I^+^ cells was calculated as the ratio of cells showing the respective staining (for MAP2 the morphology was also included) compared to the total number of cells with DAPI-stained nuclei.

## Results

### Analysis of undifferentiated ebiNSc^IDH1wt^ and ebiNSc^IDH1R132H^

**Fig 1:**
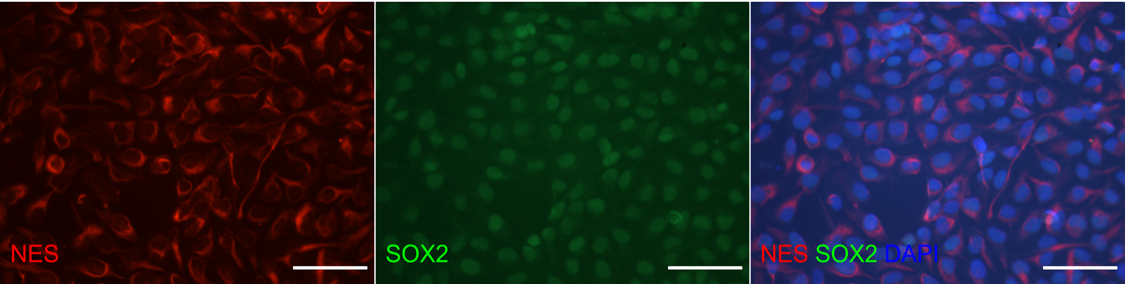
Expression of stem cell markers by undifferentiated ebiNSc. Double staining with antibodies against Nestin (red) and SOX2 (green) showing expression of neural stem cell markers in the same cells. Each image was taken at magnification 400x; scale bars mark 50μm.

**Fig 2:**
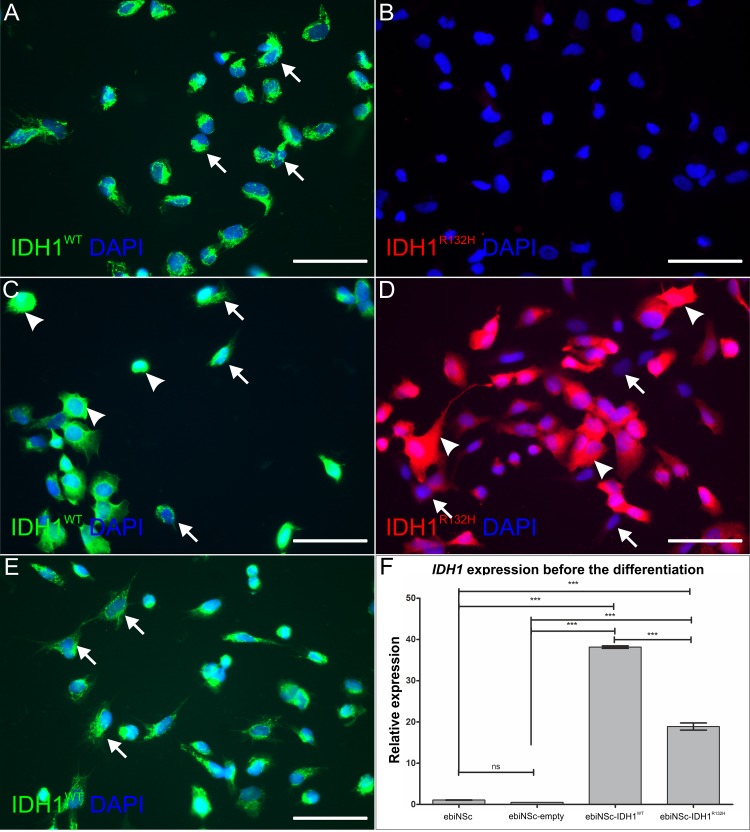
IDH1 expression in ebiNSc. **(A)** Endogenous wild type IDH1 expression in the non-transuded ebiNSc controls. Arrows mark the characteristic punctuate expression pattern. **(B)** Lack of the endogenous IDH1^R132H^ expression in the non-transuded ebiNSc controls. **(C)** Expression of wild type IDH1 in ebiNSc transduced with wild type gene, ebiNSc^IDH1wt^. Arrows mark the punctuate pattern characteristic for the endogenous IDH1. Arrowheads mark the strong, diffuse pattern of the induced expression. **(D)** Expression of IDH1^R132H^ in ebiNSc transduced with the mutant gene, ebiNSc^IDH1R132H^. Arrows mark cells lacking the induced expression. Arrowheads mark the strong, diffuse pattern of the induced expression. **(E)** Expression of endogenous wild type IDH1 in ebiNSc transduced with the empty vector, ebiNSc^empty^. Arrows mark the characteristic punctuate expression pattern. Each image was taken at magnification 400x; scale bars mark 50μm. **(F)** *IDH1* (non-mutation-specific) expression at the mRNA level in the four cultures Error bars indicate SEM. Statistical significance calculated by One-way ANOVA with Tukey's post-comparisons test. ***, p<0.005; ns, not significant.

**Fig 3:**
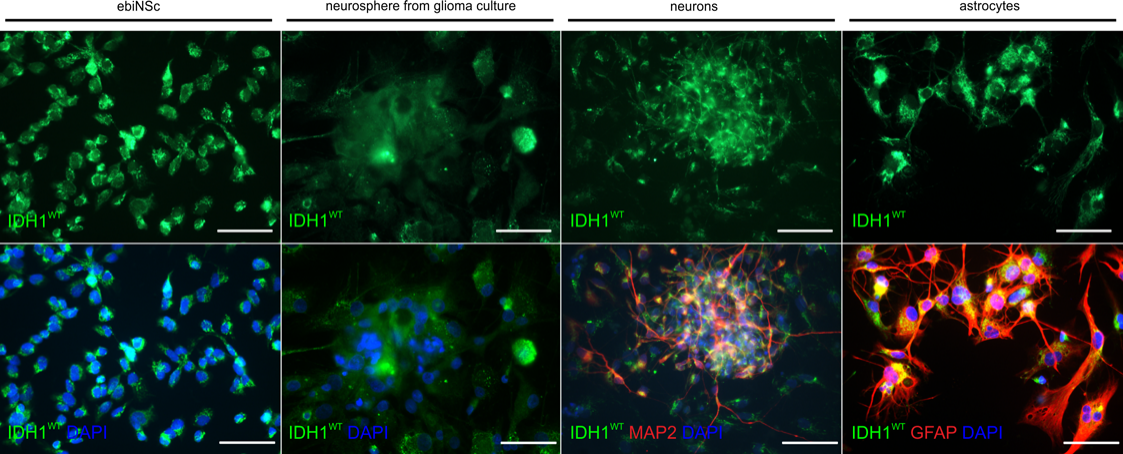
Expression of wild type IDH1 protein in different cell types. Endogenous wild type IDH1 expression in the ebiNSc, neurospheres derived from glioblastoma primary culture, neurons and astrocytes. Each image was taken at magnification 400x; scale bars mark 50μm.

The ebiNSc, presenting confirmed neural stem cell phenotype (Fig 1), were transduced with lentiviral expression vectors containing the appropriate cDNA sequence (wild type, IDH1^WT^ or mutant, IDH1^R132H^) under the control of EF1α promoter or were transduced with the empty lentiviral vector as a control. Immunocytochemical analysis was conducted to verify the expression of the transgenes. As expected, the non-transduced ebiNSc lacked the mutant IDH1 and expressed the endogenous wild type IDH1 at a low level with a characteristic punctate pattern (Fig 2A and 2B). Analysis of ebiNSc transduced with wild type gene revealed that a small population of the ebiNSc^IDH1wt^ cells had a characteristic punctate pattern of expression, while the majority of cells had a strong, diffuse, cytoplasmic expression resulting from the introduced transgene (Fig 2C). Analogously, in ebiNSc^IDH1R132H^ two subpopulations were also observed, the majority with strong exogenous expression of the mutant gene, IDH1^R132H^, and the minority with only the endogenous expression of wild type IDH1 (Fig 2D). The cells transduced with empty vector did not differ from the non-transduced ones (Fig 2E). The RT-PCR analysis using primers that do not discriminate wild type *IDH1* from its mutant confirmed overexpression of constructs as intended (Fig 2F). Additional immunocytochemistry analysis of wild type IDH1 was performed to compare its expression level in the ebiNSc-based model to that observed in normal neural cells. Generated induced neural stem cells, neurons and astrocytes all demonstrated evident expression of endogenous wild type IDH1 (Fig 3). Similar pattern of IDH1 expression was shown in neurospheres derived from glioblastoma primary cultures (Fig 3).

### Analysis of neuronal and astrocytic differentiation

**Fig 4:**
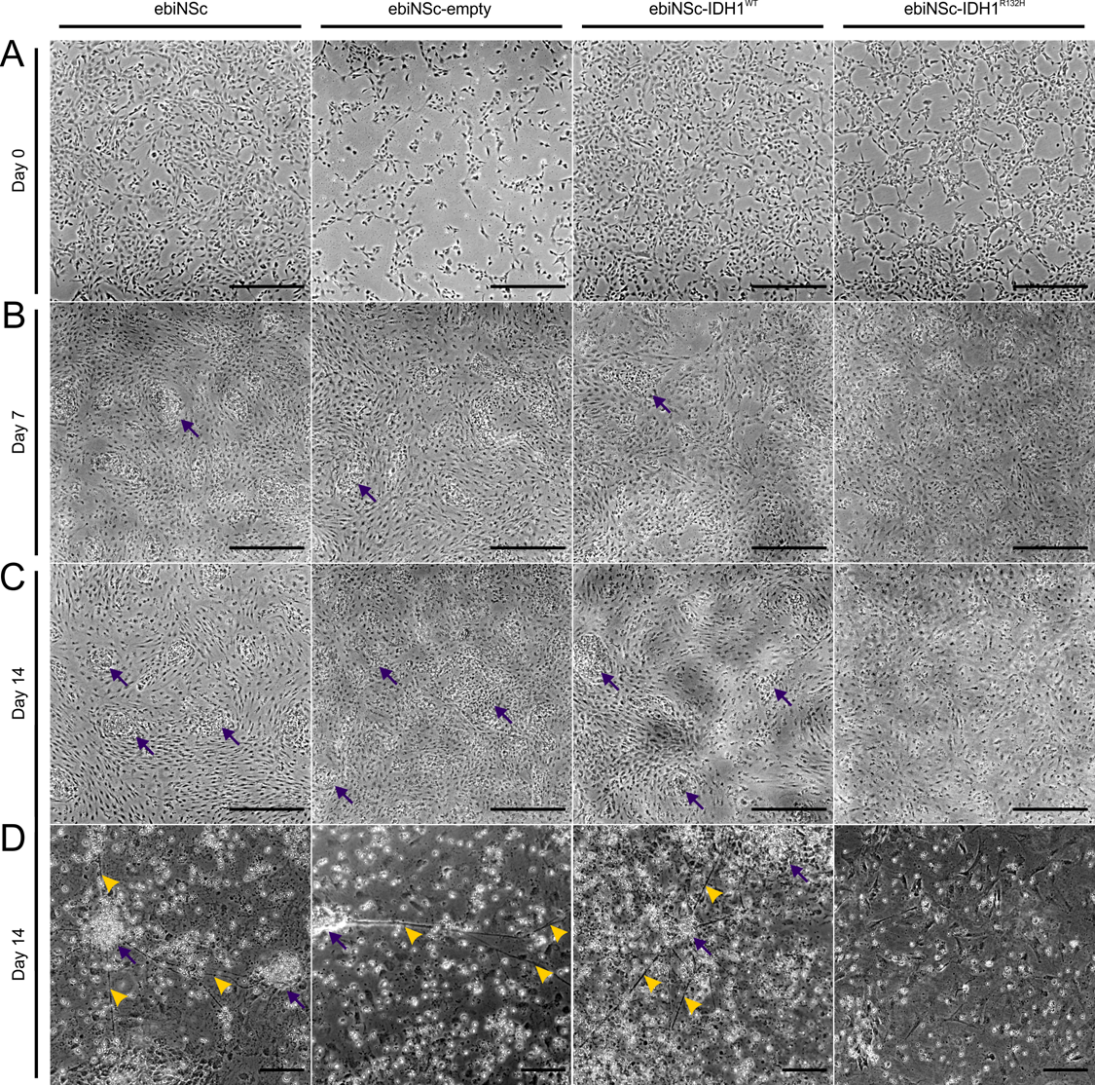
Cultures of ebiNSc expressing IDH1^R132H^ retained morphology of undifferentiated cells. **(A)** Micrographs showing morphology of ebiNSc expressing IDH1^R132H^ along with control cultures before differentiation (day 0). No evident morphological differences between ebiNSc^IDH1R132H^ and control cultures were visible on day 0. Cells were incubated in differentiation medium and photographed every 7 days in culture. **(B)** Morphology after 7 days of differentiation. Early cluster formation is visible in ebiNSc, ebiNSc^empty^, ebiNSc^IDH1wt^. **(C)** Morphology after 14 days of differentiation. More advanced cluster formation is visible in ebiNSc, ebiNSc^empty^, ebiNSc^IDH1wt^, but not in ebiNSc^IDH1R132H^. Photomicrographs taken at magnification 40x. **(D)** Morphology after 14 days at higher magnification (magnification 100x, scale bars mark 50μm). The characteristic features of the differentiating cells are marked with arrows (cell clusters) and arrowheads (network-like connections between clusters). For better readability, the light microscopy images in this figure were contrast-enhanced.

**Fig 5:**
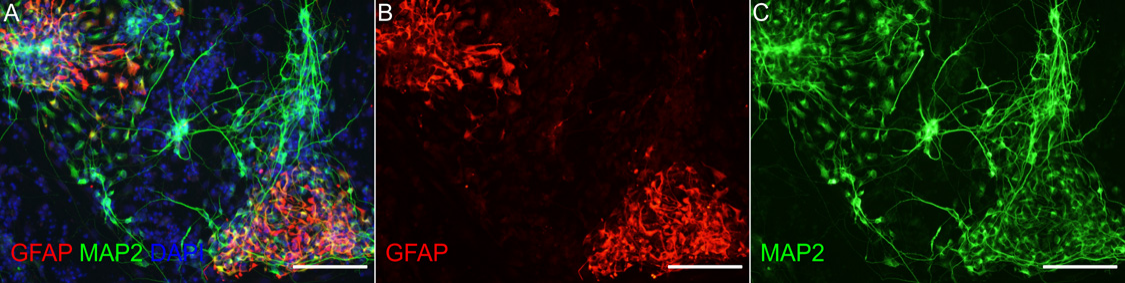
Expression of glial and neuronal markers by differentiated ebiNSc. Double staining with antibodies to GFAP (red) and MAP2 (green) after 14 days of differentiation of non-transduced ebiNSc showing populations of astrocytic and neuronal cells. Photomicrographs demonstrate **(A)** Merged image of GFAP (red), MAP2 (green) and DAPI (blue) showing populations of GFAP and MAP2 positive cells with some overlap between the two markers; DAPI was used to estimate the percentage of cells positive for GFAP and MAP2; **(B)** GFAP (red); **(C)** MAP2 (green). The images at 200x magnification, scale bars mark 100μm.

**Fig 6:**
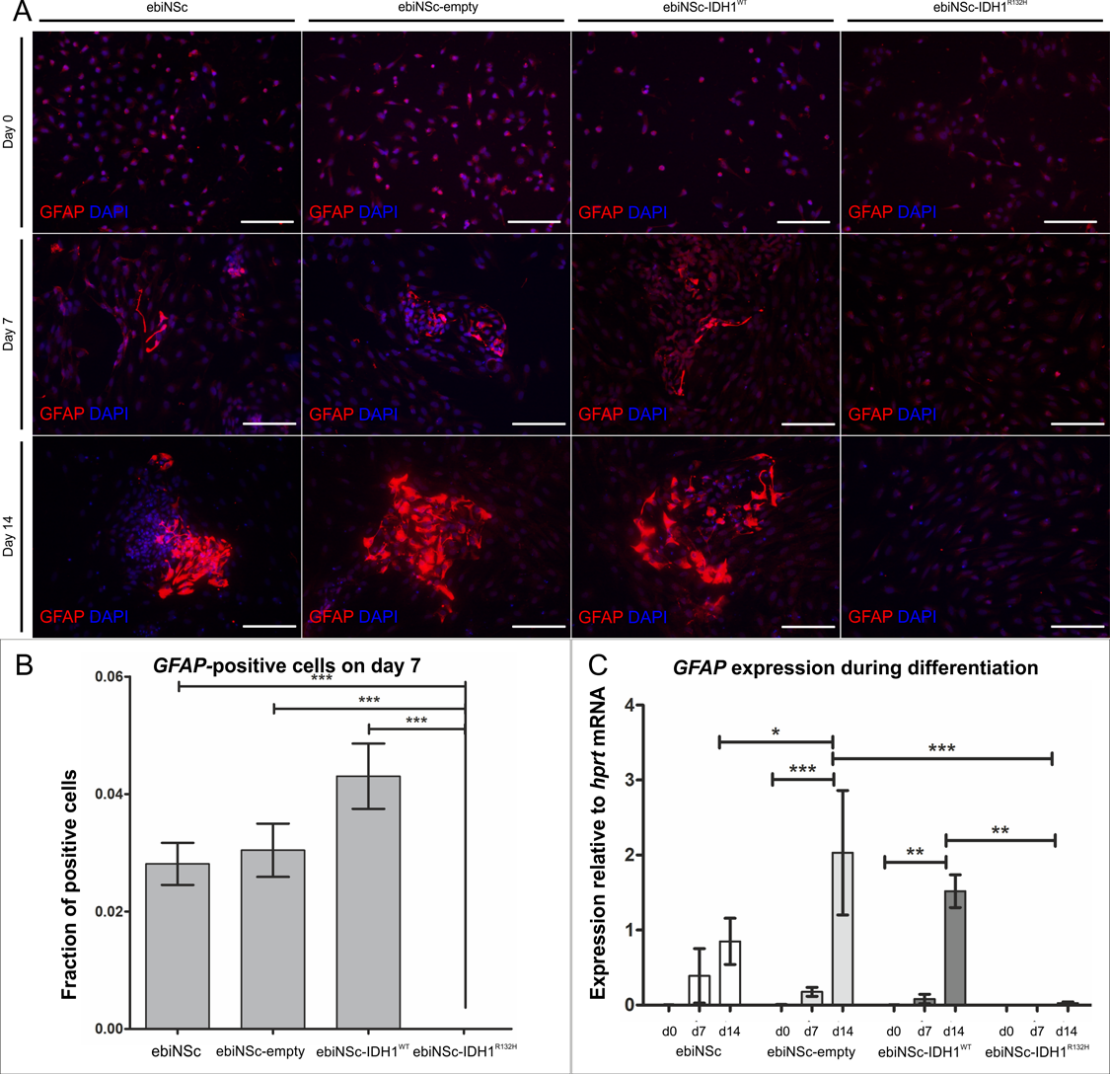
Impaired astrocytic differentiation of ebiNSc expressing IDH1^R132H^. **(A)** Immunocytochemical characterization of GFAP expression after 0, 7 and 14 days of differentiation in ebiNSc cultures expressing IDH1^R132H^ compared to control cultures (magnification 200x, scale bars mark 100μm). GFAP-positive cells are present after 7 days in ebiNSc, ebiNSc^empty^ and ebiNSc^IDH1wt^ cultures. No GFAP-positive cells are visible in ebiNSc^IDH1R132H^ after either 7 or 14 days. **(B)** Graph demonstrating the percentage of GFAP-positive cells after 7 days of differentiation in ebiNSc expressing IDH1^R132H^ and control cultures. Error bars indicate SEM. Statistical significance calculated by Kruskal-Wallis with Dunn’s multiple comparison test. ***, p<0.005. **(C**) Graph demonstrating the quantitative analysis of *GFAP* expression at the mRNA level after 0, 7 and 14 days of differentiation (d0, d7, d14) in ebiNSc expressing IDH1^R132H^ and control cultures. Error bars indicate SEM. Statistical significance calculated by Two-way ANOVA with Bonferroni’s post-comparison test. *, p<0.05; **, p<0.01; ***, p<0.005; ns, not significant.

To determine whether ebiNSc transduced with mutant IDH1 could differentiate into mature neural lineage cells, we examined the differentiation into astrocytes and neurons compared to controls. In all four cultures of ebiNSc the differentiation was induced by culturing the cells in Neurobasal Medium supplemented with B-27. The differentiation process was monitored by the observation of cellular morphology and analysis of marker expression of astrocytic (GFAP) and neuronal lineage (MAP2 and Synapsin I) at protein and mRNA levels. After 7–14 days of differentiation ebiNSc showed morphological characteristics of differentiated neural cells including formation of cell clusters and intercellular networks in all cultures, except for ebiNSc^IDH1R132H^ (Fig 4). We then stained the cells with neuron specific antibody to MAP2 and monoclonal antibody to GFAP to identify astrocytes. A double staining against GFAP and MAP2 confirmed the presence of astrocytic and neuronal cell populations in differentiated cultures on day 14 (Fig 5). When we cultured cells in differentiation medium, the proportion of cells that expressed the differentiation markers increased progressively over time. While the proportion of GFAP-positive cells increased significantly after 7 days in culture in non-transduced ebiNSc, ebiNSc over-expressing IDH1^WT^ and ebiNSc transduced with empty vector, it was absent in the ebiNSc expressing the mutant protein (Fig 6A and 6B). The proportion of cells that expressed GFAP greatly increased after 14 days of differentiation and it constituted the majority at this time point (Fig 6A). By contrast, in ebiNSc^IDH1R132H^ culture, GFAP-positive cells were not detected during the whole course of the differentiation experiment (Fig 6A). Analogous results were obtained with mRNA analysis of *GFAP* expression (Fig 6C).

**Fig 7:**
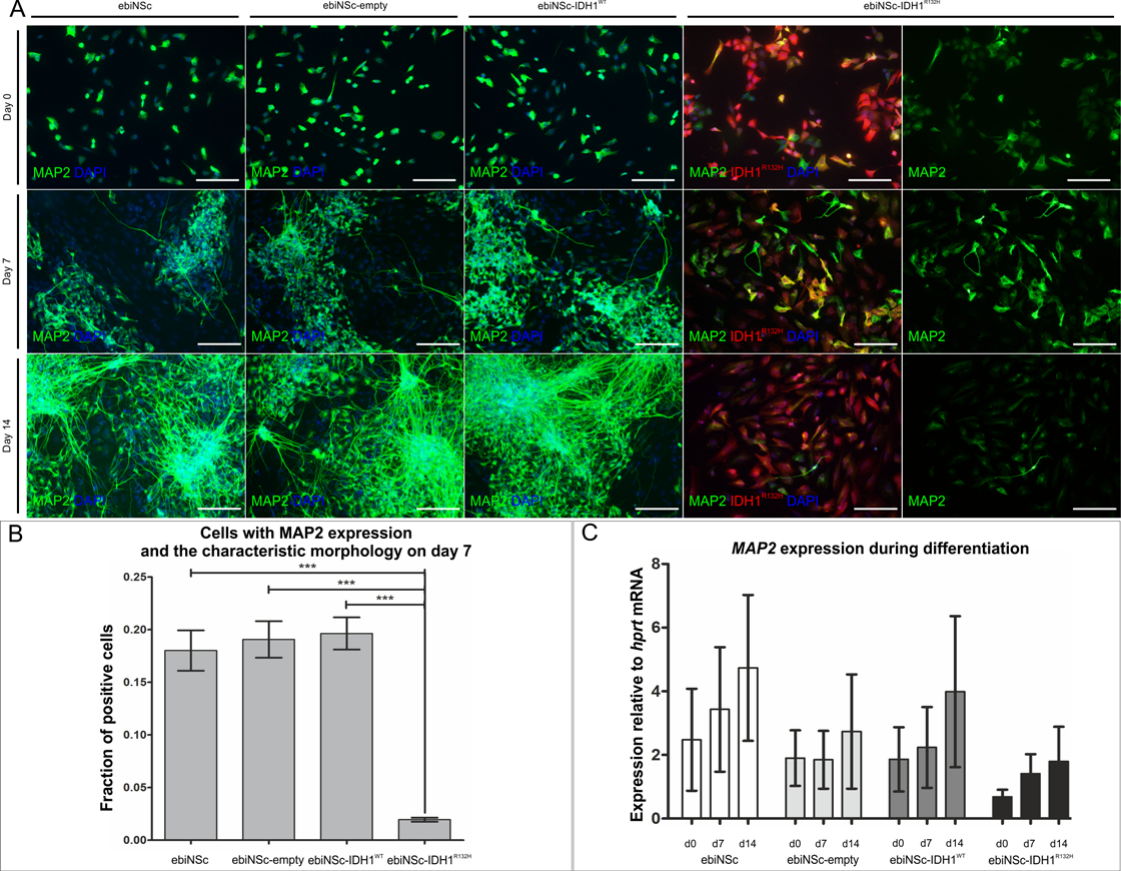
Impaired neuronal differentiation of ebiNSc expressing IDH1^R132H^. **(A)** Immunocytochemical characterization of MAP2 expression after 0, 7 and 14 days of differentiation in ebiNSc cultures expressing IDH1^R132H^ compared to control cultures (magnification 200x, scale bars mark 100μm). Fraction of MAP2-positive cells is present before the differentiation. The early clusters of MAP2-positive cells are visible after 7 days in ebiNSc, ebiNSc^empty^ and ebiNSc^IDH1wt^ controls. No MAP2-positive clusters and a low fraction of MAP2-positive cells are visible in ebiNSc^IDH1R132H^ after either 7 or 14 days. **(B)** Graph demonstrating the percentage of MAP2-positive cells with the elongated morphology after 7 days of differentiation in ebiNSc expressing IDH1^R132H^ and control cultures. Error bars indicate SEM. Statistical significance calculated by Kruskal-Wallis with Dunn’s multiple comparison test. ***, p<0.005. **(C)** Graph demonstrating the quantitative analysis of *MAP2* expression at the mRNA level after 0, 7 and 14 days of differentiation (d0, d7, d14) in ebiNSc expressing IDH1^R132H^ and control cultures. Error bars indicate SEM. Two-way ANOVA analysis did not reveal any statistical significance between samples, however, the trend can be appreciated.

**Fig 8:**
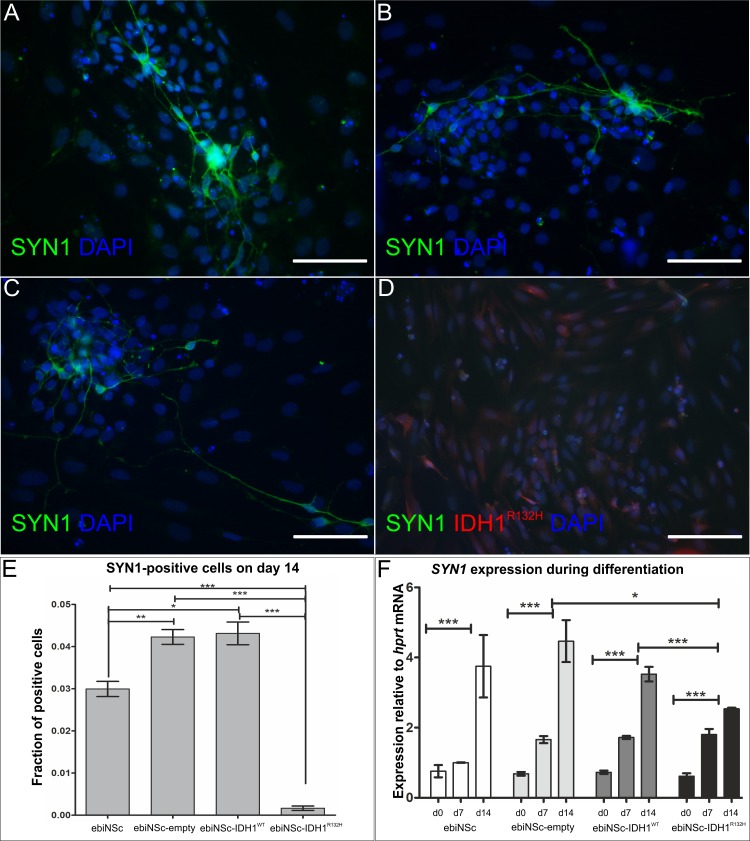
Immunocytochemical characterisation of Synapsin I expression in differentiating cells. Synapsin I expression after 14 days of differentiation in ebiNSc cultures expressing IDH1^R132H^ compared to control cultures**. (A)** Synapsin I (SYN1) expression in ebiNSc. **(B)** Synapsin I expression in ebiNSc^IDH1wt^. **(C)** Synapsin I (SYN1) expression in ebiNSc^empty^ **(D)** Staining with antibodies to Synapsin I (green) and IDH1^R132H^ (red) in ebiNSc^IDH1R132H^. No SYN1-positive cells are visible. All images at magnification 200x, scale bars mark 100μm. **(E)** Graphs demonstrating the percentage of SYN1-positive cells after 14 days of differentiation in ebiNSc expressing IDH1^R132H^ and control cultures. Error bars indicate SEM. Statistical significance calculated by Kruskal-Wallis with Dunn’s multiple comparison test. *, p<0.05; **, p<0.01; ***, p<0.005. **(F)** Graph demonstrating *SYN1* expression at the mRNA level after 0, 7 and 14 days of differentiation (d0, d7, d14) in ebiNSc expressing IDH1^R132H^ and control cultures. Error bars indicate SEM. Statistical significance calculated by Two-way ANOVA with Bonferroni’s post-comparison test. **, p<0.01; ***, p<0.005; ns, not significant.

A population of cells in all ebiNSc cell lines expressed *MAP2* before the differentiation (day 0). However, these cells did not demonstrate any distinguishable morphology of mature neuronal cells. After 7 days of differentiation, we observed a significant increase in the number of MAP2-positive cells and the characteristic elongated morphology in the non-mutant ebiNSc cells (Fig 7A). By contrast, in ebiNSc^IDH1R132H^ the number of MAP2-positive cells with the characteristic morphology of neuronal cells on day 7 of differentiation was significantly lower than in the other cultures (Fig 7B). This differentiation tendency continued till the end of the experiment and cells in ebiNSc^IDH1R132H^ culture retained an undifferentiated state after 14 days of differentiation. *MAP2* expression at the mRNA level generally reflected the trend; however, the differences were not statistically significant (Fig 7C). Additionally, on day 14 we analysed the expression of Synapsin I (SYN1), a neuron-specific protein (Fig 8). Similarly to the MAP2 analysis, the number of SYN1-positive cells was significantly higher in ebiNSc, ebiNSc^IDH1wt^ and ebiNSc^empty^ than in ebiNSc^IDH1R132H^ (Fig 8E). The expression at the mRNA level showed a similar trend (Fig 8F).

### Apoptosis of neural stem cells and their derivatives

**Fig 9:**
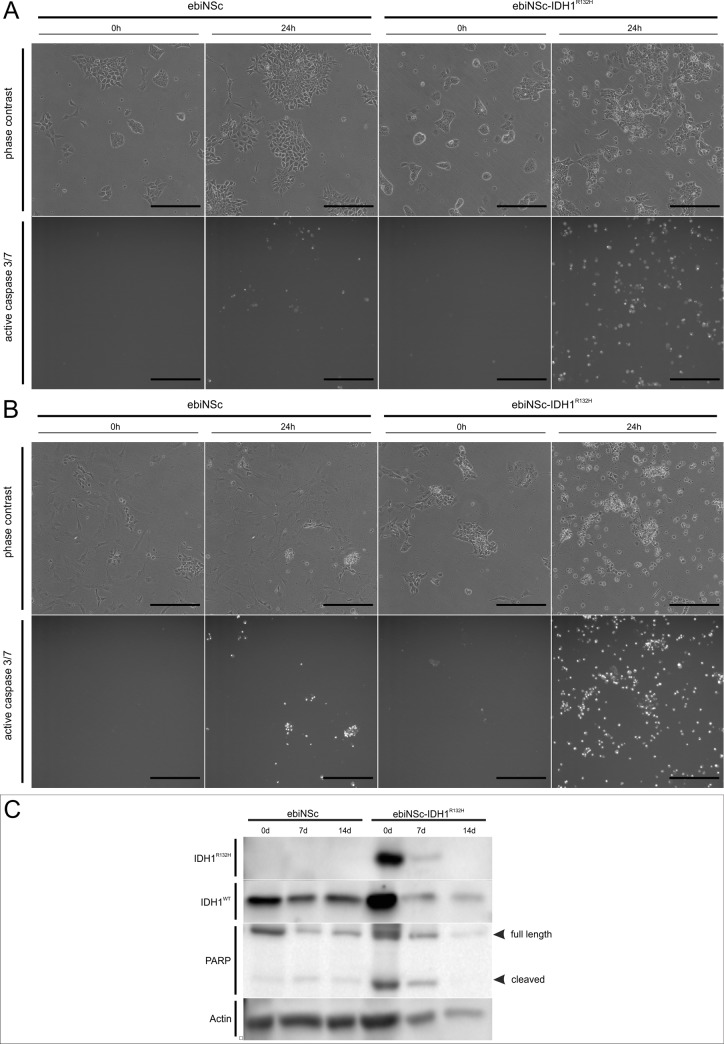
IDH1^R132H^ increases apoptosis susceptibility of induced neural stem cells and their derivatives. **(A)** Micrographs showing apoptotic cells in control ebiNSc and ebiNSc^IDH1R132H^ cultured in non-differentiating medium with synthetic reporter of Caspase 3/7 activity. Increased apoptosis visible in IDH1^R132H^-expressing cells. **(B)** Apoptotic cells in control ebiNSc and ebiNSc^IDH1R132H^ after 7 days of differentiation. Increased number of apoptotic cells was observed in ebiNSc^IDH1R132H^ cells compared to differentiating control ebiNSc. Experiment was conducted in a manner similar to that described in A. Each image was taken at magnification 100x, scale bars mark 50μm. **(C)** Western Blot analysis for PARP in non-transduced ebiNSc and ebiNSc^IDH1R132H^ under differentiating and self-renewing conditions. In case of undifferentiated cells (0d), more cleaved form of PARP was observed in ebiNSc^IDH1R132H^ than in control. The decrease in PARP level during differentiation of ebiNSc^IDH1R132H^ may be correlated with reduced total protein amount as indicated by actin.

To determine whether the IDH1^R132H^ mutation negatively affects cell survival of induced neural stem cells, cells ectopically expressing this construct and non-transduced cells were cultured in standard medium used for cell propagation (non-differentiating medium) with synthetic reporter of Caspase 3/7 activity and subjected to real-time observation for 2 days. As expected from previous reports, the activity of the synthetic reporter was more prominent in the IDH1^R132H^-expressing ebiNSc compared to the non-transduced control ebiNSc (Fig 9A). To investigate the association between differentiation and apoptosis, analogous observation was performed on cells on the 7th day of differentiation procedure. Increased number of apoptotic cells was observed in ebiNSc IDH1^R132H^ cells compared to differentiating control ebiNSc (Fig 9B). Furthermore, the number of apoptotic cells undergoing differentiation was much higher than that observed in propagating ebiNSc^IDH1R132H^. To verify these observations, western blot analysis detecting cleaved form of PARP, a well-characterized caspase-3 substrate, was performed. In case of undifferentiated cells, more cleaved form of PARP was observed in ebiNSc^IDH1R132H^ than in control, which is consistent with the results obtained during the real-time observation (Fig 9C). Similar dependence was observed at every stage of the differentiation process. Interestingly, the increased abundance of cleaved form of PARP was also observed for differentiated control cells. The decrease in PARP level during differentiation of ebiNSc^IDH1R132H^ may be correlated with reduced total protein amount (as indicated by low level of actin), resulting from robust apoptosis and precluding the visualization of all protein bands of interest. Nevertheless, sequencing analysis of nucleic acids isolated from ebiNSc^IDH1R132H^ derivatives confirmed expression of mutated gene after 7 and 14 days of differentiation (data not shown). In conclusion, both approaches indicate that neural stem cells expressing IDH1^R132H^ were more susceptible to apoptosis than normal neural stem cells. Also, similar dependence was demonstrated between eiNSc^IDH1R132H^ derivatives and ebiNSc derivatives.

## Discussion

The high frequency of mutations in the isocitrate *IDH1* gene in diffuse gliomas indicates its importance in the process of gliomagenesis [6]. IDH1^R132H^ mutation, frequently defined as oncogenic, results in loss of the normal function and acquisition of the neomorphic activity converting α-ketoglutarate (αKG) to 2-hydroxyglutarate (2HG) [8,11]. Both, αKG and 2HG affect the epigenetic status of cells *via* chromatin modifying enzymes [12], which, in turn, is the key element in the process of differentiation [18]. Other mechanisms of dioxygenase inhibition were also reported in gliomas, *e*.*g*. hypermethylation of the TET2 promoter [19]. One of the effects of the TET protein inhibition is the reduction of the genomic content of 5-hydroxymethylcytosine (5hmC), which may be an intermediate step during the 5mC (5-methylocytosine) demethylation [20]. Higher levels of 5hmC were shown both in the more differentiated compartments of foetal brains [21] and in the more differentiated glial tumours [20]. Also, low 5hmC levels were reported to be related with poor outcomes of malignant glioma patients [21]. Gliomas with *IDH1* mutations have the neural stem cell phenotype and the adequate histone methylation profile [22]. One of the mechanisms of impaired differentiation of glioma cells is the reduced expression of CRABP2 (transcription factor related to retinoic acid signalling), which results from the general hypermethylation [23]. The origin of gliomas is suspected to locate among the glial progenitor cells and IDH1/2 mutations presumably initiate gliomagenesis [13].

Previously, we have analysed IDH1^R132H^ mutation in glioma cells, revealing that role of this protein is associated with high level of expression [16]. Here, we analysed the effects of IDH1^R132H^ mutation on human neural stem cells generated from induced pluripotent stem cells, under differentiating and propagating conditions. Hitherto, IDH1^R132H^ has only been analysed in tumour cells, differentiated astrocytes, or stem cells of non-neural lineage [22,24–30]. Therefore, novel *in vitro* models are required to study the effects of *IDH1* gene mutations. The increased concentrations of 2HG induced *via* either IDH1/2 mutation or its direct addition to the cell culture were shown to increase the proliferation and growth of astrocytes in soft agar [24] as well as propagate accumulation of histone changes and induced Nestin expression [22]. In a mesenchymal model, mutant IDH1 was reported to induce chondrogenic differentiation, while inhibiting the osteogenic differentiation *via* histone modifications [25]. Another group reported the similar impairment of osteogenic differentiation by 2HG, however, there was no impact on adipocytic differentiation, while the chondrogenic differentiation was variably influenced [26]. Of note, they reported no effect on chondrocytes, indicating that the observations from differentiated cell models cannot be directly translated to stem cell models [26]. In a hepatoblastic model, mutant IDH2 inhibited the differentiation *in vitro* and caused an aberrant response to hepatic injury *via* progenitor cell hyperplasia in a mouse model [27]. In a haematological model (erythroleukemia), IDH2 mutation caused methylation changes, which were successfully reversed by its inhibitor [18]; similarly, it impaired the EPO-induced differentiation, which was restored upon mutant IDH2 inhibition [28]. Moreover it has been shown that transient transfection with wild type IDH2 caused an increase in 2HG production [29]. By contrast, in this study we showed that in our induced neural stem cell model the induction of wild type IDH1 expression did not hinder cell differentiation, which should reflect such an increase.

The mouse conditional knock-in models with mutant IDH1 induced in Nestin-expressing cells were reported to die directly after birth, while the analogous GFAP-induced models survived slightly longer, but did not develop gliomas during their lifespan [30]. Accumulation of 2HG, high HIF1α levels and impaired collagen maturation were observed in IDH1^R132H^-expressing cells, however, instead of the expected elevated ROS levels, its lower concentrations were observed [30]. Another group reported a lack of astrocytic differentiation in an *in vivo* mouse model following replantation of IDH1^R132H^-transduced neurospheres [22]. Similarly to our studies, virtually no astrocytic differentiation was observed in ebiNSc transduced with the mutant gene, while the percentage of neuronal cells was significantly decreased.

In order to ensure the reliability of the results, we employed four independently generated populations of ebiNSc and analysed the impact of overexpression of either wild type or mutant (R132H) IDH1 on the astrocytic and neuronal differentiation in comparison to cells transduced with empty vector and the non-transduced cells. Apart from the markers of stemness, all of the cells presented expression of wild type IDH1 at levels comparable to normal neural cells and neurospheres derived from glioblastoma primary culture. The characteristic morphological changes were observed only in the non-transduced cells, or the cells with induced IDH1^WT^ expression and empty vector. The immunocytochemical stainings confirmed those observations, with the relative number of differentiated cells significantly reduced in the IDH1^R132H^ expressing cells. Cells positive for GFAP, MAP2 or Synapsin I were almost completely absent from the culture in contrast to the control cell lines. With the relatively low differentiation efficiency provided by the protocol, the assessment at the mRNA level is only partially informative as it offers insight into the entire population of cells. Still, the mRNA levels of *GFAP* and *Syn1* on day 14 of differentiation reflected the findings on the protein level, whilst *MAP2* mRNA levels trended in the same direction, although not reaching statistically significant difference. This may partially result from the fact that the expression of *MAP2* is also detected in a fraction of undifferentiated ebiNSc. Such low expression levels of differentiation markers in neural stem cells have already been reported [31].

Our findings in regards to the effect of IDH1^R132H^ mutation on cell survivability are in line with previous reports [32,33]. We have observed elevated apoptosis of cells expressing the mutant form of the protein in comparison to non-transduced cells. This observation was true for both investigated states: during propagation as well as during differentiation. However, the extent of apoptosis was not as dramatic in cells undergoing propagation when compared to the cells undergoing differentiation. It is worth mentioning that apoptosis is a natural process accompanying neural stem cell functional specialization during the development of central nervous system [34]. Therefore, the apoptosis observed during ebiNSc differentiation is consistent with the current state of knowledge on developmental biology. It can be suggested that the elevated apoptosis observed in IDH1^R132H^ expressing cells is likely to be a cumulative effect of pro-apoptotic processes occurring during differentiation and cell-intrinsic influence of the mutant protein.

The elevated apoptosis associated with IDH1^R132H^ construct introduces a counterintuitive aspect, when considering the mutation in question as oncogenic, in particular its key role in initiation of tumorigenesis suggested in the literature [7]. Nevertheless, recent data demonstrated the pro-apoptotic role of IDH1^R132H^ in stabilized cancer cell lines. Daming Cui and colleagues strongly suggested that the IDH1^R132H^ serves as tumor suppressor in human glioma by negatively regulating Wnt/β-catenin signaling [35]. To this end, we postulate the search for another genetic or epigenetic change preceding IDH1 mutations during gliomagenesis.

Previously, IDH1^R132H^ was considered as differentiation blocking agent [22, 27]. Inhibition of differentiation is thought to be crucial for cancer biology, however it is expected to be a consequence of mechanisms supporting cancer cells symmetric divisions [36]. Mechanisms augmenting the susceptibility of IDH1^R132H^-expressing cells toward apoptosis, in differentiating or in self-renewing conditions, cannot be classified as inhibiting differentiation, but rather impairing it.

Investigation of *IDH1* mutation is associated with substantial challenges due to inability to culture *in vitro* primary glioma cells showing IDH1^R132H^ [37]. It questions the reliability of induced IDH1^R132H^ cell models, however no better ones have been introduced so far. Gliomas are apparently developed from GFAP-positive neural stem cells, and remain GFAP-positive after differentiation inhibition [38]. To this end, GFAP-positive neural stem cells model seems to be more accurate to investigate the role of IDH1 mutations in gliomagenesis. Unfortunately, protocols for derivation of such cells from iPSc are not available yet.

Summarizing, in spite of some issues, the presented model is attractive for the investigation of the impact of mutations in particular genes on astrocytic and neuronal differentiation of stem cells. This model offers numerous possibilities for further development and application. Apart from the *IDH1*, other tumour suppressors and oncogenes associated with gliomagenesis (as described by Ohgaki *et al*. [13]) may be further studied using our model. An important advantage of this model in comparison to others is the possibility to analyse this process with or without the sequentially added elements of the neoplastic molecular context. Such an approach may eventually lead to understanding the causes and mechanisms of glioma emergence as well as indicate new therapeutic targets. In addition, it may be used for the preclinical studies on IDH1/2 inhibitors, which gradually progress into the clinical trial phase (*e*.*g*. NCT02428855). Thus far, the effects of such inhibitors were analysed in a primary oligodendroglioma culture, where they induced histone demethylation and re-expression of target genes, which further caused the loss of stem-like properties and astrocytic differentiation [39]. Similar results were obtained with decitabine (DNMT inhibitor) in an analogous model [40]. Intriguingly, in mouse xenografts, high doses of mutant IDH1 inhibitor caused tumour growth inhibition, methylation, and expression changes as well as astrocytic differentiation; however, even low doses were sufficient for an equivalent growth impairment despite no effect on the epigenetic or expression profile [39]. Therefore, other functions of the mutant isocitrate dehydrogenase or 2-hydroxyglutarate, beyond the described methylation induction, may be expected and require further studies.

In summary, this is the first study of the IDH1^R132H^ influence on the *in vitro* neural differentiation of human neural stem cells. Our study shows that IDH1^R132H^ enhances apoptosis susceptibility impairing neural differentiation. Detected pro-apoptotic role of IDH1^R132H^ which is still considered as oncogene, raises many question on its role in gliomagenesis.
